# Effects of Task Constraints and Experience Level on Adaptations of Running in Young Basketball Players: Interlimb Coordination, Symmetry and Variability

**DOI:** 10.1177/00315125261418373

**Published:** 2026-01-24

**Authors:** Shahab Parvinpour, Mohsen Shafizadeh, Poorya Maleki, Keith Davids

**Affiliations:** 1 Faculty of Physical Education and Sport Sciences, 145440 Kharazmi University, Tehran, Iran; 2 College of Health, Wellbeing and Life Sciences, 7314 Sheffield Hallam University, Sheffield, UK

**Keywords:** movement patterns, constraints, running gait, coordination

## Abstract

Running is a functional movement skill in team sports that requires timing, coordination and symmetry in swing, stance and flight phases, continuously shaped by constraints of specific performance contexts (e.g., running with and without a ball). The aim of this study was to examine how interlimb coordination, symmetry and temporal variability in running patterns are regulated by changes in task design and player’s experience level. Participants in this study were novice (N = 12; 2 months practice experience) and trained/intermediate (N = 12; 12 months practice experience) basketball players who ran at their typically preferred pace around a basketball court in two conditions: running only and running whilst dribbling a basketball. Running gait was assessed by recording mean temporal measures of stance, swing and flight phases, along with measures of temporal variability, bilateral symmetry and phase coordination index in the two conditions. Findings of this study revealed that changes in task constraints, from running without the ball to dribbling, resulted in less functional interlimb coordination, increased asymmetry and variability, regardless of player’s experience level. In conclusion, these findings suggest that interlimb coordination, symmetry and variability are temporal running gait parameters that become attuned to required adaptations to specific task constraints (e.g., when dribbling a ball), regardless of experience level. These findings have important implications for the design of training tasks for development of running speed while coordinating actions in team sports.

## Introduction

Running is a functional movement skill that is developed in early childhood between ages 1.5 and 2 years and reaches a mature form at the age of 12 years (Goodway et al., 2019). According to Dynamic System theory (DST), in locomotor development across the lifespan, many system control parameters (e.g., strength, balance, speed, perception of information) are shaped by interactions of personal and environmental constraints that limit or enable transitions from one stable, functional movement pattern (e.g. walking) to another (e.g. running) (Thelen et al., 1987). Synchronisation and interlimb coordination are common features of oscillatory movements in running characterised by a bounded temporal relationship and couplings developed between the legs (Scott Kelso et al., 1981; Scott Kelso et al., 1983). The phase coordination of two limbs defines the organisation of a movement pattern (order parameter). Changes to adapting coordination patterns (locomotor activity) emerge through transitions in a key information variable (control parameter) such as movement frequency (speed) at critical values for each individual (Warren Jr., 1990). According to concepts of the DST, learning or adapting movement pattern is a process of transition to more functional actions by destabilising a stable attractor state (functional coordination pattern) through changes in system control parameters (Haken et al., 1985; Kelso, 1984).

This conceptual framework may be used to understand how to design more representative training tasks in sports to enhance skill adaptation during learning (Pinder et al., 2012). To exemplify, running is a common requirement in many sports and a key concern is how best to enrich the functionality of coordination patterns when running at speed under different task constraints of competition. This issue is part of a broader challenge for sport scientists and practitioners challenged with designing physical training tasks for athletes that incorporate integrated coordination of movement skills (see for example Burnie et al., 2022). In many situations in invasion sports such as basketball, running is highly dynamic and complex, requiring a functional integration of locomotor actions like jumping or gait, with other relevant actions. The need for functional, integrated coordination of different actions increases task complexity by adding more active components to be re-organised.

In basketball, the ability to run with and without the ball is important and emphasised in the early phase of sport skills development. In many competitive contexts, players learn to run with the ball (dribbling skills) for implementing offensive tactics. They also learn to use running patterns for fast transitions from offence to defence (without the ball) to restrict space and prevent scoring opportunities for opponents (Gréhaigne et al., 2012). Thus, the ability to re-organise running coordination patterns from an on-the-ball state to an off-the-ball state, and vice versa, is paramount for the player’s individual and collective functionality in team performance.

Adaptations of movement patterns in sport emerges through practice, the environment and the task (Handford et al., 1997; Newell, 1986). Previous studies have shown how manipulation of task constraints, as a part of practice organisation, may lead to re-organisation of running coordination patterns (Bailey et al., 2018; Hafer et al., 2016; Okudaira et al., 2021). For example, adaptations in coordination patterns were observed when running at different speeds, and on different surfaces and slopes. Adaptations which emerged included more anti-phase coordination patterns when running on steeper slopes (Okudaira et al., 2021) and altered segment coordination and reduced coordination variability in mid-late stance at higher speeds (Hafer et al., 2016). In basketball dribbling, Heidarnia et al. (2022) showed that the integration of simulated sport visual attention and dual-task performance tasks, during the run-to-cut phase, elicited variations in hip-ankle, hip-knee, knee-knee, and knee-ankle couplings. Performance adaptations have also been noted following practice task manipulations involving presence (or lack of presence) of a defender when attackers run to cross a ball in football (Orth et al., 2014). Robalo et al. (2021), investigated training effects on basketball dribbling and showed that professional players displayed significantly lower levels of variability in wrist movements, but had a significantly higher value of variability in horizontal shoulder movements.

It seems that the adaptation of cyclic movement patterns through practice has only been studied in cyclical motions in low complexity contexts, with participants running. There have been no attempts to investigate effects on locomotion gait patterns when athletes are challenged to perform an integrated skill such as dribbling with a ball while running. In many team sports that require cyclic locomotor movements, such as dribbling or running with the ball, motor adaptations are more complex and need to be continuously adjusted due to the constraint of integrating hand-leg coordination, as well as perceptual information from the trajectory of a ball in motion. Thus, the aim of this study was to examine how interlimb coordination, symmetry and temporal variability in running patterns become adapted to changes in practice task constraints (running vs. dribbling) and experience levels in young basketball players.

## Methods

### Participants

The participants of this study were 24 young basketball players from novice and intermediate groups. The sample size was calcuated with G*power software at 0.80 power of test, 95% confidence interval and effect size (0.30). The novice (N = 12; mean age = 13.41 ± 2.02years; height = 159.58 ± 13.14 cm; body mass = 53.75 ± 11.55 kg) and trained/intermediate (N = 12; mean age = 13 ± 2.75years; height = 163.8 ± 16.03 cm; body mass = 58.7 ± 16.55 kg) players were recruited non-randomly from a basketball club. The players and coaches after receiving information about the purpose of the study and its methodology voluntarily took part in this study. Participants were males and free of any injuries during testing and had achieved basic levels of skill in essential basketball techniques. All participants were able to dribble continuously at speed without losing control of the ball over a long period of time, based on coaches’ assessments. The novice group had 2 months of experience in basketball, whereas the trained group had 12 months of basketball experience. They had only worked with the same coach 2-3 sessions per week and 90 minutes each session and never took part in any other basketball club training sessions. They received information about the aims of the study and co-signed (with parents) the consent forms that was approved by a local university ethics committee.

### Materials

To measure participant running gait under the different task manipulations, two low-mass (<3 g) inertial measurement unit (IMU) sensors (MetaMotion R; MBIENTLAB Co., San Francisco, CA), were used, with a 3D accelerometer, 3D gyroscope, and magnetometer. The sensors were equipped with Bosch Sensortec (Stuttgart, Germany) to combine the measurements of the accelerometer, gyroscope, and magnetometer and provide the orientation vector. The sensors were attached to the medial–distal part of the right and left tibia to reduce the effect of soft tissue vibration. The unobtrusive sensors were secured using double-sided tape and Velcro adjustable straps (Presco, Swindon, United Kingdom). The axis of each sensor was aligned with the vertical axis of the lower leg while the participant was standing (X: superior-inferior; Y: mediolateral; Z: anterior-posterior). The sensors sampled movements at a frequency of 240 Hz. For detecting the stance phase, the gyroscope and accelerometer of the tibia sensor were synchronized. The sensor-based gait analysis have previously been used in investigations of walking and running gait events (McGrath et al., 2012; Shafizadeh et al., 2019) and reported good reliability and validity against the marker-based motion capture systems (McGrath et al., 2012). To determine the running temporal events, the initial contact and toe-off points of each leg were determined by the gyroscope of the tibia sensor, according to the methods of McGrath et al. (2012). The initial contact and toe-off point of each leg were detected when angular velocity reached its minimum value on the *y*-axis. The data were collected by the MetaBase mobile application that received the IMU signals through Bluetooth for data processing. The raw gyroscope signal was low pass filtered with a zero-phase, fifth-order Butterworth filter with a 40 Hz frequency.

### Procedure

Running data were collected on the basketball court of the club. Participants wore their training shoes and a running lane was set up that required the participants to run in an oval pathway outside of the basketball perimeter. This task setup helped them to undertake constant pace running without sudden changes in direction and speed. The test protocol included two running trials: running without the ball (running) and running whilst dribbling the ball (dribbling) at a self-paced speed. The conditions were randomly selected for the participants so that half of the participants started the running test first and others started the dribbling test first. To determine the target running pace, the participants were asked to familiarise themselves with running at their preferred speed a few times. In the dribbling condition, they were asked to use their preferred hand throughout the entire trials and not to change hand during the task. Participants were tested individually and ran for 2 minutes in each condition. For further analysis, 50 strides from each leg in straight lines running were selected.

### Data Analysis

The dependent variables in this study included: mean temporal parameters, temporal variability, phase coordination index (PCI), and leg swing asymmetry. Three phases of a running gait cycle, such as stance, swing and flight, were determined through analyses of running gait events. Dribbling tempo was defined as the ratio of steps per number of basketball bounces, with a higher number indicating fewer bounces per running step.

#### Temporal Running Parameters

Values for swing time, stance time of each leg and flight time were calculated and reported as the average and coefficient of variation (CV) from successive strides. The CV values were used as temporal variability metrics in this study.

#### Swing Asymmetry

Swing asymmetry was measured by comparing the swing times of the right and left legs in every cycle. This formula was used to quantify the asymmetry (Plotnik et al., 2007):

Asymmetry = 100 × |ln (SSwing time/LSwing time)|

SSwing and LSwing time refer to the mean value of swing time for the leg, calculated with short and long mean swing times, respectively. A higher score represents a greater temporal asymmetry between the right and left leg swing.

#### Interlimb Coordination (PCI)

The PCI provides an index of the relationship between the right and left legs. The procedure to calculate the PCI for running gait was extracted from the walking gait (Plotnik et al., 2007). Simply, the PCI is a phase difference (ϕ) between the right and left legs at every initial point of contact. Ideally, this phase difference should be anti-phase and equal to 180° (see Figure 1).

$$\Phi =360˚\times \left(T_{\text{Si}}\;–\;T_{\text{Li}}\;/\;T_{L\;\left(i+1\right)}-T_{\text{Li}}\right)$$

Where T_Li_ and T_Si_ denote the time of the *i*th initial contact point of the legs with the long and short average swing times.

**Figure 1.**
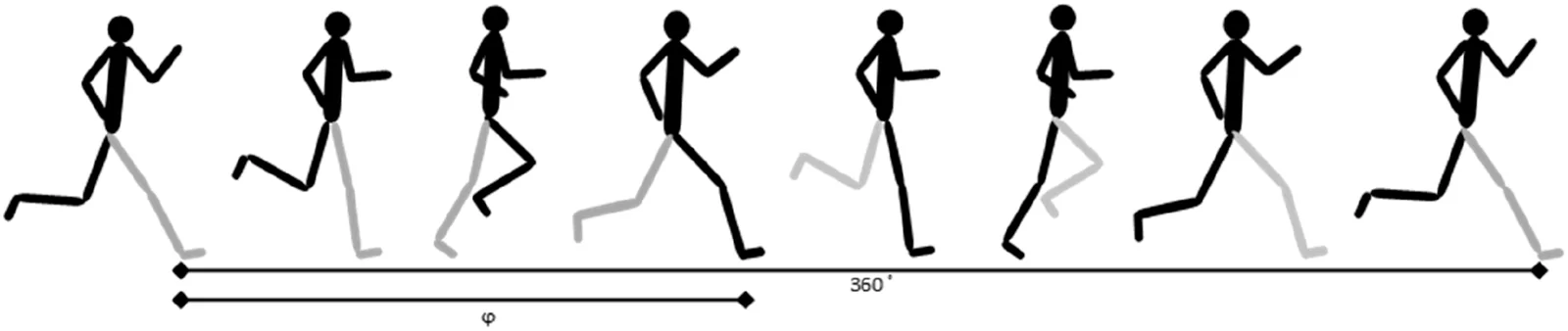
An illustration of phase determination of one leg stride with respect to the running cycle determined by the other leg

Then, the accuracy and consistency levels of ϕ were calculated. The level of accuracy is the amount of phase difference from 180° (ϕ_Abs):

ϕ_Abs=abs (ϕi−180)

The level of consistency (ϕ_CV) is calculated by a difference of each phase value from the mean value. The PCI is the sum of the phase accuracy and phase consistency values as below:

PCI = ϕ_CV + PCI_Abs

where

PCI_Abs=100×(ϕ_Abs/180)

A higher PCI score represents a higher phase difference between the right and left swing legs, thus indicating less functional interlimb coordination.

We also calculated the task difficulty cost on PCI and asymmetry index based on dual-task cost paradigm (Plummer & Eskes, 2015) through this equation:

Difficulty cost = dribbling-running/running;

where positive values indicate the increased task difficulty cost from running to dribbling.

A 2-way mixed analysis of variance (group × task) with a repeated measure on the last factor was used to statistically analyse differences between mean values of the dependent variables between the novice and trained groups and between running and dribbling tasks. A Pearson correlation coefficient was used to examine relations between the dribbling tempo and the PCI in the dribbling task. The confidence interval was set at 95% (two-tailed). To measure the variance of task and training status on temporal changes, partial eta squared (ƞ^2^) tests were used; a value closer to 1 indicates the high contribution of the independent variable to changes in the dependent variable.

We pilot tested the sensor with 5 participants to calcuate intra-rater reliability within 30 seconds trials (test-retest). The Pearson correlation coefficients were 0.88 (PCI), 0.90 (CV) and 0.93 (asymmetry).

## Results

### Dribbling Tempo

The average number of steps per ball bounce (tempo) was 2.13 (±0.3), and the lowest and highest tempo rates were 1.1 and 3, respectively. The number of bounces per swing leg (Right/Left%) was 40:60 (±17) and the lowest and highest percentage values were 15:85 and 75:25, respectively.

### Interlimb Coordination (PCI)

Interlimb coordination was significantly greater and less functional (F_1,22_ = 7.83, p = 0.003, ƞ^2^ = 0.26) in the dribbling task (5.9 ± 2) than in the running task (4.71 ± 1.9). The main effect of group and the interaction of group and task were not statistically significant (F <1, p = 0.14). A lower PCI value in dribbling was related to increased inaccuracy (ϕ_Abs) in interlimb coordination relative to anti-phase (F_1,22_ = 9.05, p = 0.001, ƞ^2^ = 0.29; dribbling = 8.58 ± 3.17; running = 6.26 ± 3.46) and not inconsistency (ϕ_CV) in successive strides (see Figure 2).

**Figure 2.**
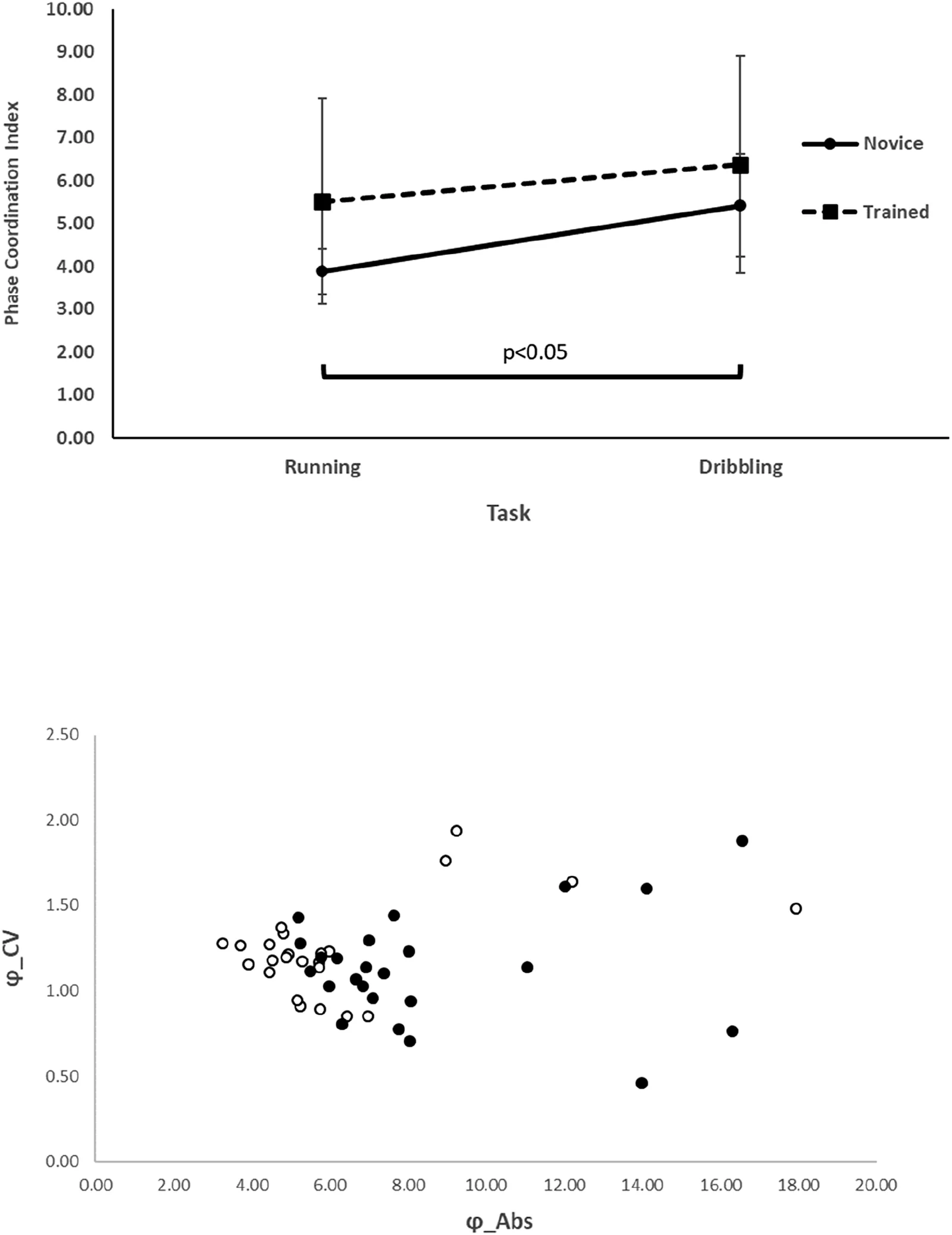
The mean (SD) values of PCI in different groups and tasks (top). The values of ϕ_Abs and ϕ_CV (bottom) in the dribbling (black circles) and running (white circles). The deviation from the origin indicates the increased inaccuracy (ϕ_Abs) in interlimb coordination relative to the anti-phase pattern and inconsistency (ϕ_CV) in successive cycles

The task difficulty cost in PCI in novice and trained groups was 0.40 (±0.3) and 0.24 (±0.52), respectively, a difference that was not statistically significant (t < 1, p = 0.23).

There was an inverse and significant relationship (r = −0.44, p = 0.018) between dribbling tempo and the PCI in dribbling.

### Asymmetry

The running asymmetry was significantly greater (F_1,22_ = 4.51, p = 0.01, ƞ^2^ = 0.17) in the dribbling task (3.18 ± 3) than in the running task (1.65 ± 0.70). The main effect of group and the interaction of group and task were not statistically significant (F <1, p = 0.32).

Task difficulty cost in asymmetry in novice and trained groups was 2.14 (±3.87) and 0.25 (±0.61), respectively, and there were no between-group differences (t < 1, p = 0.29). See Figure 3 for the group and task differences in the asymmetry index.

**Figure 3.**
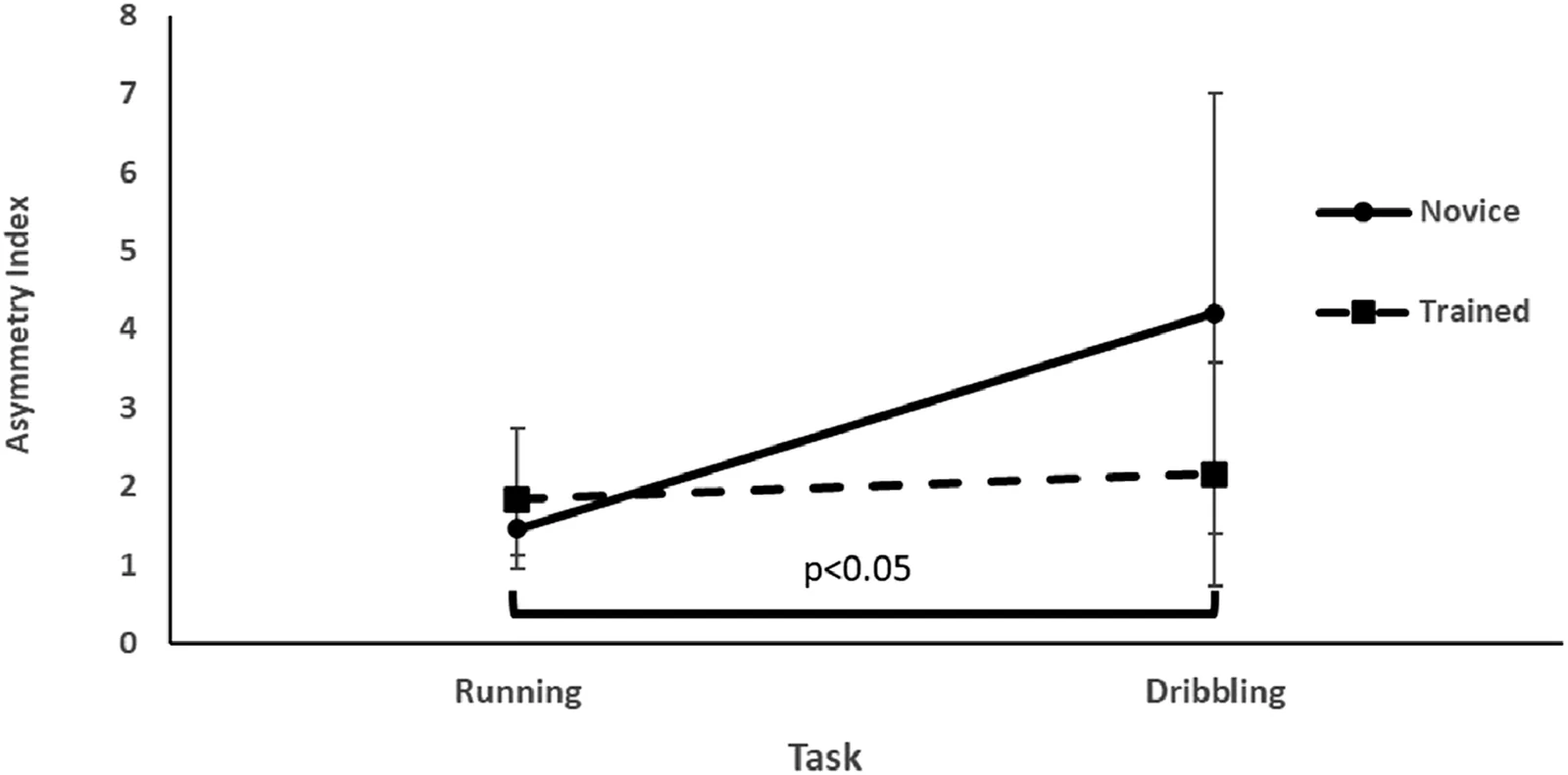
The mean (SD) values of the asymmetry index in different groups and tasks

### Temporal Variability

Swing time variability in the right leg (F_1,22_ = 6.36, p = 0.009, ƞ^2^ = 0.22) and the left legs (F_1,22_ = 6.23, p = 0.008, ƞ^2^ = 0.22) was significantly greater in the dribbling task (R:10.75 ± 8.9; L:11.33 ± 9.1) than in the running task (R:6.15 ± 4.36; L:6.7 ± 3.95). The main effect of group and the interaction of group and task were not statistically significant (F <1, p = 0.13).

Stride time variability in the right (F_1,22_ = 6.16, p = 0.007, ƞ^2^ = 0.22) and the left legs (F_1,22_ = 5.91, p = 0.006, ƞ^2^ = 0.21) was significantly greater in the dribbling task (R:6.24 ± 4.8; L:6.1 ± 4.93) than in the running task (R:3.7 ± 2.32; L:3.62 ± 1.78). The main effect of group and the interaction of group and task were not statistically significant (F <1, p = 0.33).

The flight time variability was significantly greater (F_1,22_ = 8.81, p = 0.01, ƞ^2^ = 0.28) in the dribbling task (32.58 ± 21.78) than in the running task (21.37 ± 11.22). The main effect of group and the interaction of group and task were not statistically significant (F <1, p = 0.22).

The main effect of group, task and the interaction of group and task were not statistically significant for stance time variability (F <1, p = 0.18).

### Temporal Parameters

The main effect of group, task and the interaction of group and task were not statistically significant in any temporal measures (F <1, p = 0.31).

The means and standard deviations (SD) of running temporal measures are presented in Table 1.

**Table 1.** Means and Standard Deviations of Running Gait Parameters in Different Groups and Tasks

Parameters	Novice		Trained	
	Running	Dribbling	Running	Dribbling
*PCI components*				
Phi_Abs	4.98 (1.14)	7.7 (2.01)	7.55 (4.01)	9.48 (4.4)
Phi_CV	1.12 (0.16)	1.15 (0.25)	1.33 (0.32)	1.1 (0.38)
*Temporal Measures*				
R. stride time (sec)	0.660 (0.04)	0.644 (0.04)	0.649 (0.06)	0.647 (0.06)
L. stride time (sec)	0.660 (0.04)	0.644 (0.04)	0.648 (0.06)	0.647 (0.06)
R. swing time (sec)	0.232 (0.02)	0.215 (0.02)	0.237 (0.04)	0.225 (0.03)
L. swing time (sec)	0.296 (0.05)	0.304 (0.02)	0.286 (0.02)	0.300 (0.03)
R. stance time (sec)	0.256 (0.02)	0.250 (0.02)	0.222 (0.02)	0.244 (0.04)
L. stance time (sec)	0.262 (0.02)	0.266 (0.02)	0.230 (0.02)	0.250 (0.04)
Flight time (sec)	0.101 (0.02)	0.080 (0.02)	0.121 (0.04)	0.090 (0.04)
*Temporal Variability*				
R. stride time (CV%)	2.86 (0.61)	7.03 (6.1)	4.54 (3.1)	5.45 (3.38)
L. stride time (CV%)	2.86 (0.72)	6.94 (6.1)	4.38 (2.21)	5.26 (3.4)
R. swing time (CV%)	4.43 (1.13)	11.3 (10)	7.86 (5.66)	10.22 (7.8)
L. swing time (CV%)	4.73 (1.33)	11.27 (9.9)	8.86 (4.73)	11.38 (8.5)
R. stance time (CV%)	5.1 (0.98)	11.1 (13)	17.92 (16.2)	20.88 (25)
L. stance time (CV%)	5.9 (1.06)	11.7 (13.8)	18.08 (14.7)	21.75 (26.3)
Flight time (CV%)	17.28 (5.31)	31.77 (22.2)	25.46 (14.1)	33.38 (22.31)

## Discussion

The aim of this study was to examine whether interlimb coordination, symmetry and temporal variability in running patterns are affected by task manipulations (from running to dribbling) and experience levels of young basketball players. Findings revealed that dribbling a basketball whilst running can significantly lead to less functionality in interlimb coordination and increased asymmetry and temporal variability in the running patterns, regardless of experience levels of participants. These differences could be indicative of adaptation of running gait patterns when ball dribbling was integrated in the task and the first hypothesis regarding differences between running and dribbling tasks in coordination, symmetry and variability was accepted. We tested two types of adaptations in this study: short-term and long-term. The first form of adaptation, short-term, emerged as a result of changes in the task manipulations. Our results supported the fact that increased difficulty in the skill resulted in participants adapting interlimb coordination and asymmetry and increasing temporal variability. The increases in the cost of running pattern in coordination by 0.24-0.40 times and in symmetry by 0.25-2.14 times were because of performing an additional task (controlling the bouncing ball by hand) during running that required a different adaptation in the motor system. Because the participants of this study had adequate proficiency in speed dribbling, they adapted running patterns by slight changes in anti-phase coordination between legs and temporal asymmetry and variability. The technical adaptations were creating arm-leg coordination so that after approximately every 2 steps, 1 arm movement emerged (average dribbling tempo was 2.13). This style of dribbling influenced the interlimb coordination index so that participants with fewer arm movements (fewer ball bounces and higher tempo) displayed a lower value of PCI and vice versa. Lower PCI values observed in this study may indicate that the participants of this study were still learning to adapt their running gait to the task constraint of dribbling with a ball. In addition, this coordination pattern was used through a bilateral and variable strategy in which the ball bouncing time was coincident with the swing phase of both legs (right-left 40:60).

These findings are aligned with data from previous studies which examined adaptations of running coordination patterns and exploitation of movement variability as task constraints altered (Bailey et al., 2018; Hafer et al., 2016; Okudaira et al., 2021). Similar observations of skill adaptation have been reported in studies of basketball dribbling (Heidarnia et al., 2022) which included dual-tasking during the run-to-cut phase, eliciting different lower limb movement patterns. In football, it was also shown that dribbling a football on a treadmill increases physiological cost, compared to running without dribbling (Reilly & Ball, 1984).

In our study, task constraints were manipulated by adding an additional task (arm-ball bouncing) that could have interfered with gait patterns when dribbling a ball. An adapted coordination pattern emerged from the functional system formed by the dribbler with the ball. The greater variability and adaptations to coordination pattern when dribbling may have been indicated of participants exploring the functional system states to shape emergent coordinative patterns. The main challenge here is either maintaining the stable coordination pattern or exploring other movement solutions in order to adapt to the different task demands (Neumann, 2016). According to tenets of DST, observed locomotor system adaptations due to task constraints manipulations in this study may have emerged by exploiting inherent self-organisation tendencies in the form of movement system fluctuations in gait attractor states (anti-phase coordination, 180°) to facilitate movement adaptations (Haken et al., 1985; Kelso, 1984). By harnessing interlimb fluctuations (see Figure 2, bottom), participants were able to use higher values of interlimb coordination as a functionally adaptive strategy when the task was changed from running to dribbling. It seems that a functional strategy to dribble at a self-paced speed in novice and intermediate players was to maintain stride-to-stride interlimb variability, exploiting deviations (7.7-9.48°) from the stable anti-phase coordination pattern. Because the main aim of the running task whilst dribbling was not affected by these changes (successful performance and no dribbling errors as eligibility criterion in this study), we can conclude that all changes in interlimb coordination, symmetry and variability were at functional levels for locomotor adaptations of a foundational movement pattern (running) applied in a sport-specific context (running while dribbling a ball).

The second hypothesis regarding the effects of experience levels on coordination, symmetry and variability was not accepted. This hypothesis was related to the idea of a longer-term process of adapting gait to the task constraint of dribbling a ball. Our results showed that extra practice opportunities to train basic basketball skills had little effect on running patterns. These findings are not consistent with some previous research outcomes on this issue. Some previous studies have reported higher coordination variability in expert performers in basketball dribbling (Robalo et al., 2021) and swimming (Seifert et al., 2010), whereas there are some reports that have not revealed any advantages of more experience (McDermott et al., 2003; Nikodelis et al., 2005). One issue in the current study concerns the differences between experience levels of the two treatment groups: ten months may not have been enough to elicit adaptations to key characteristics of gait when dribbling and running without the ball. This area requires more studies with greater differences between experience levels (e.g., years rather than months) in order to fully understand practice effects on gait adaptations.

Another related explanation for the lack of group differences is that the attractor state for running (anti-phase coordination of the legs) was more greatly affected by development processes, without enough room for the learning process to have a great effect. Because running patterns reach a mature, stable state of organisation at the age of 12 years in many typically developed children, additional practice had no effects on the coordination pattern, symmetry and variability in these groups of children. The main role of practice is to stabilise a to-be-learned movement pattern (Zanone & Kelso, 1992, 1997) and when the pattern is stabilised by at the time scale of development, extra practice opportunities may need more practice time (>10 months) in order to enhance adaptive functionality of gait when dribbling a ball.

The findings of this study have some implications to practice. The complex nature of limbs organisation when carrying a ball implies that a representative design of skills practice may need to be carefully considered for greater performance effectiveness, as advocated elsewhere (Burnie et al. (2022). These ideas can be applied to the way that practice tasks can be designed in team games to help learners adapt and re-organise their functional movement patterns (e.g., running and jumping in isolation) in order to contextualise performance to competitive constraints (running with a ball and jumping to intercept a ball).

This study had some limitations. First, the skill level was defined based on the years of play rather than expertise. Future investigations on this issue could be improved by redefining the skill level based on quality of performance rather than time spent practising (McKay et al., 2022). Second, despite data collection in the real basketball court, the context was not truly representative of the basketball performance. The speed of the dribbling action in this study was self-paced and constant which may differ in performance contexts in which pace is changed. In addition, the running pattern around the basketball field was similar to distance running. While there are occasions when players dribble at a self-selected speed in basketball, often performance contexts dictate that running can be complex, variable, and not completely internally paced. These are issues for future research after confirmation in this study that running is adapted in team sport like basketball, when dribbling with a ball.

In conclusion, the findings of this study showed that short-term adaptations of functional movement patterns in team sport skills are implemented to adapt to task constraints, resulting in some re-organisation of key running parameters such as interlimb coordination, bilateral symmetry and temporal variability.
